# Editorial: Improving cancer chemotherapy through pharmacogenomics: a research topic

**DOI:** 10.3389/fgene.2015.00195

**Published:** 2015-06-03

**Authors:** Luis A. Quiñones, Kuen S. Lee

**Affiliations:** ^1^Laboratory of Chemical Carcinogenesis and Pharmacogenetics, Clinical and Molecular Pharmacology Program, Faculty of Medicine, Instituto de Ciencias Biomédicas, University of ChileSantiago, Chile; ^2^Department of Surgery, Hospital del Salvador, University of ChileSantiago, Chile

**Keywords:** pharmacogenomics, pharmacogenetics, polymorphism, genetic, oncology, antineoplastic agents

It is well known that the efficacy and safety of chemotherapeutic drugs show substantial individual and/or population variability that can be explained, to a great extent, by genetic factors in pharmacokinetics and pharmacodynamics (Quiñones et al., 2008; Roco et al., 2012; Izar et al., 2013; Kessler et al., 2014; Patel, 2015; Unger et al., 2015). In the context of anticancer drugs, polymorphisms in genes encoding drug-metabolizing enzymes, drug transporters and drug targets influence the pharmacokinetics and pharmacodynamics, have been shown to affect clinical outcomes (Binkhorst et al., 2015; Newman et al., 2015). Several chemotherapeutic agents can be more harmful to normal tissues than the targeted tumor, and their use may result in tumor cell resistance, toxicity and occasionally secondary neoplasia. Current practices for the dosing of therapeutic agents could be improved through the understanding of gene variation that impacts the life of the drug inside the body. In order to better predict a patient's predisposition to treatment complications and poor outcomes, it is essential to consider all candidate loci influencing response to the chemotherapeutic agents. This requires better understanding of the metabolic pathways for activation or inactivation of these drugs, drug interactions, age and gender sensitivities, and the impact of ethnicity and environmental factors. As an added benefit, a more rational use of expensive treatment drugs, together with actions to minimize patient toxic events and their consequences, would dramatically reduce medical costs.

Pharmacogenomics is an emerging field focused on genetic variations relevant to drug action (Evans and Relling, 2004; Maxmen, 2011; Mayer, 2014; Zhou et al., 2015). Solid data concerning allele variants, haplotypes and their effects on gene expression, applied to the use of chemotherapy regimens' design and dosing has the potential to enhance treatment outcomes. Some of the potential polymorphic candidate genes include the CYP isozymes, transferases, dehydrogenases, deaminases, reductases, ABC transporters, drug receptors and DNA repair enzymes (Agundez, 2004; Bruhn and Cascorbi, 2014; Roco et al., 2014).

With this Research Topic we aim to present an updated overview of the current trends in the experimental genetic approaches worldwide, and discuss future directions in cancer pharmacotherapy. The scope of this issue is to collect evidence to conceive chemotherapy tailored to each patient.

In this special edition we have five manuscripts: four reviews and one original article. Filipski et al. (2014) discuss the use of observational studies, as well as the use adaptive trials and next generation sequencing to achieve the required level of evidence for clinical implementation. They propose to move the research field toward retrospective analyses, large population studies using Electronic Medical Records, and mechanism-based evidence to propel progress in the field. In addition, embracing new methods, such as next generation sequencing and pharmacogenomic adaptive clinical trials, will enrich our knowledge about tumor biology, and change the clinical approaches for cancer treatment. They highlight that up-to-date pharmacogenomic studies tend to focus on either somatic or germline mutations in isolation, but to truly optimize clinical care both genomes need to be integrated into clinical decision making. Agúndez et al. (2014) review the development of clinical practice recommendations or guidelines for the clinical use of pharmacogenomics data. They suggest that this is an essential issue for improving drug therapy, particularly for drugs with high toxicity and/or narrow therapeutic index, such as anticancer agents. The few available clinical practice guidelines for translating Pharmacogenomics are insufficient for its application in the use of anticancer therapy. The application of available guidelines, further implementation with clinical feedback, plus a combination of genomics and phenomics is urgently required. Roco and co-workers explore the possibility for prediction of the efficacy and safety of cisplatin treatment using a pharmacogenomic approach, and focus primarily on DNA reparation and Glutathione S-Transferases (GSTs) enzymes. They conclude that there is a potential utility for pharmacogenetics in therapeutic schemes employing cisplatin. This could be addressed through collaborative efforts to study molecular characteristics of drug treatment in order to develop a genetic panel for this context.

Finally, T. Ishikawa's mini-review and the original article of Zocche et al. (2015) describe the use of two relevant genes in oncology as prognostic biomarkers. Ishikawa (2014) focuses on genetic polymorphism in the *NRF2* gene, especially in the future for individualized cancer lung cancer treatment. The author supports the role of this gene as a mediator of cancer cell proliferation and drug resistance, and highlight the −617C>A change in the anti-oxidant response element (ARE)-like loci as relevant in the attenuation of the positive feedback loop of transcriptional activation of *NRF2*, leading to reduced NRF2 protein levels. As a consequence, it is considered cancer cells will become more sensitive to therapy and less aggressive than cancer cells harboring the wild type allele. Therefore, genetic polymorphisms in *NRF2* might serve as prognosis markers for cancer therapy. Finally, Zocche et al. (2015) investigated the clinical application and the Global Impact of KRAS mutation patterns in 5-fluorouracil regimens (FOLFOX and FOLFIRI). The study investigated the progression-free survival and overall survival in colorectal cancer patients receiving FOLFOX treatment with respect to KRAS mutations. They conclude that G12D KRAS mutation was significantly associated with a poor prognosis.

We proudly present the current Research Topic and believe it contributes evidence for the better understanding of the genetic basis in individual differences in response to antineoplastic drugs, as well as suggesting potential biomarkers for treatment prognosis.

## Conflict of interest statement

The authors declare that the research was conducted in the absence of any commercial or financial relationships that could be construed as a potential conflict of interest.
